# Data management practice of health extension workers and associated factors in Central Gondar Zone, northwest Ethiopia

**DOI:** 10.3389/fdgth.2024.1479184

**Published:** 2024-11-06

**Authors:** Mequannent Sharew Melaku, Lamrot Yohannes

**Affiliations:** ^1^ Department of Health Informatics, Institute of Public Health, University of Gondar, Gondar, Ethiopia; ^2^ Department of Environmental and Occupational Health and Safety, Institute of Public Health, College of Medicine and Health Science, University of Gondar, Gondar, Ethiopia

**Keywords:** data, data management practice, health extension workers, associated factors, Central Gondar Zone

## Abstract

**Introduction:**

Generating quality data for decision-making at all levels of a health system is a global imperative. The assessment of the Ethiopian National Health Information System revealed that health information system resources, data management, dissemination, and their use were rated as “not adequate” among the six major components of the health system. Health extension workers are the frontline health workforce where baseline health data are generated in the Ethiopian health system. However, the data collected, compiled, and reported by health extension workers are unreliable and of low quality. Despite huge problems in data management practices, there is a lack of sound evidence on how to overcome these health data management challenges, particularly among health extension workers. Thus, this study aimed to assess data management practices and their associated factors among health extension workers in the Central Gondar Zone.

**Method:**

An institution-based cross-sectional study was conducted among 383 health extension workers. A simple random sampling method was used to select districts, all health extension workers were surveyed in the selected districts, and a structured self-administered questionnaire was used for data collection. The data was entered using EpiData version 4.6 and analyzed using STATA, version 16. Bivariable and multivariable binary logistic regression analyses were executed. An odds ratio with a 95% confidence interval and a *p*-value of <0.05 was calculated to determine the strength of the association and to evaluate statistical significance, respectively.

**Results:**

Of the 383 health extension workers enrolled, all responded to the questionnaire with a response rate of 100%. Furthermore, 54.7% of the respondents had good data management practices. In the multivariable logistic regression analysis, being a married woman, having good data management knowledge, having a good attitude toward data management, having 1–5 years of working experience, and having a salary ranging from 5,358 to 8,013 Ethiopian Birr were the factors significantly associated with good data management practices among health extension workers. The overall data management practice was poor with only five health extension workers out of ten having good data management practices.

## 1 Introduction

Healthcare information is important for effective clinical and managerial decision-making at different levels of a health system (1–3). A health information system (HIS) is one of the essential building blocks of a health system aimed at producing valid health knowledge to support clinical, public health, and management decision-making. Data management is one of the six components of HIS and it is defined as the effective coordination of people, materials, and procedures to gather, process, compile, and interpret data, and display the results using a table or graph for evidence-based decision-making. It is a system that provides the accurate, reliable, valid, and timely information necessary for the improvement of public health services, requiring effectiveness and efficiency through better management of data at all levels of implementation (2).

Health information systems in most of the world are inadequate and do not provide the needed managerial support (4–6). Globally, the majority of health facilities either do not submit any health reports or have no standardized format. This in turn creates problems in drawing inferences for managerial decision-making (7). Furthermore, data received from many health facilities are incomplete, inaccurate, and unrelated to the priority tasks and functions of local health personnel (4, 7).

Most health systems in developing countries have inadequate information systems with endless registers and sending out reports without receiving any feedback (1, 8). The HISs in most developing countries are inefficient, poor in quality, and are greatly affected by data unreliability resulting from inadequate collection and poor aggregation and analyses (9). Reports from sub-Saharan Africa indicate that vital health decisions are made based on crude estimates of disease and treatment burdens (9, 10).

The evidence consistently shows that poor data management significantly hinders health outcomes and decision-making processes at all levels, ranging from missed diagnoses at the patient level to inefficient resource allocation in hospitals and poor national-level health policies. The poor data management practices of healthcare professionals and health extension workers (HEWs) affect health outcomes and decision-making processes at all levels of the healthcare system. The impact of poor data management skills starts in individual healthcare and extends to the national level. At the individual level, it affects continuity of care, produces inaccurate and incomplete data, and leads to incorrect diagnoses and treatment, which in turn leads to death or lifelong complications for the individual (11–14). More importantly, poor data management skills significantly impact healthcare organizations by leading to the inefficient allocation of resources such as staff, medications, and equipment, hindering the effective monitoring of health facility performance and leading to a lack of accountability and underperformance in health outcomes. Without reliable data, facilities cannot measure key performance indicators (KPIs) (15–17). Finally, poor healthcare data management impacts national-level decisions. At the national level, it leads to flawed national health policies, as decision-makers lack the necessary evidence to guide policy formulation and intervention strategies. In addition, poor data management hinders effective responses to health emergencies, such as disease outbreaks (18–20).

In Ethiopia, the impact of poor data management on health outcomes and decision-making processes at all levels is described in the literature (21–25).

HEWs are the frontline health workforce where baseline health data are generated in the Ethiopian health system (26). However, the data collected, compiled, and reported by HEWs are unreliable and of low quality (27, 28). The Community Health Information System (CHIS) is the health information system in which community health workers/HEWs practice data management (10, 29). In Ethiopia, data quality and use remain weak, particularly among health extension workers. An assessment by the Ethiopian National Health Information System indicated that health information system resources, and data management, dissemination, and use were rated as “not adequate” among the six major components of the health system. Several HIS issues arise from the poor data management practices of health extension workers.

Several studies conducted across the world identified several factors that contribute to the quality of data management practices of healthcare professionals (30–32). Evidence shows that the HIS of Ethiopia is of low quality and is affected by a lack of knowledge, practice, and insufficient analysis skills among the health workers (1, 33–37).

Despite these issues, there is a lack of sound evidence on how to overcome these health data management challenges. Therefore, assessing the data management practices of HEWs could be a valuable resource to combat these challenges. Moreover, having information about the factors affecting the data management practices of HEWs is the primary step and base for the improvement of the CHIS, the Health Management Information System (HMIS), and overall health system performance. Thus, the aim of this study was to assess data management practices and their associated factors among HEWs in the Central Gondar Zone.

## 2 Methods

### 2.1 Study area

This study was conducted in the Central Gondar Zone in the Amhara Region. Gondar is the capital city of the Central Gondar Zone and is located 173 km from Bahir Dar and 658 km from Addis Ababa. It is divided into 19 administrative districts (4 urban and 15 rural). According to a planning and program report in the Central Gondar Zone, the total projected population is 2,370,377 of which 1,180,448 are men and 1,189,929 are women. There are 9 public hospitals, 76 public health centers, and 404 health posts. There are 940 health extension workers working in these health posts.

### 2.2 Study design and period

A facility-based cross-sectional quantitative study was conducted from 5 to 15 September 2021 in the Central Gondar Zone.

### 2.3 Source and study population

The source population was all health extension workers who were working in all the districts in the Central Gondar Zone. The study population was all health extension workers currently working in the selected woredas in the Central Gondar Zone. All health extension workers who were currently working during the study period in the selected districts in the Central Gondar Zone were included and health extension workers who were seriously ill and unable to understand the purpose of the study or who were absent during the data collection period were excluded from the study.

### 2.4 Sample size determination and sampling procedure

The sample size was calculated using the single population proportion formula, *n* = *Z*(*α*/2)^2^ · *pq*/*d*^2^. We assumed: *n* = the required sample size; *Z* = the value of standard normal distribution corresponding to *α*/2, 1.96; *p* = proportion of health extension workers who have good data management practices, 53% (considering previous study conducted in East Gojjam Zone); *q* = proportion of health extension workers who have poor data management practices, 47%; and *d* = precision, 0.05. Hence, the required sample size was calculated to be 384. After adding a 10% non-response rate, a total of 423 samples was determined. However, the total number of health extension workers in the selected five districts was 383, thus we considered 383 participants for the entire analysis. The cluster sampling technique was used to select the district to be sampled. Accordingly, 5 out of 15 districts (33%) were selected using a simple random sampling technique. The selected districts were East Dembia (91), Gondar Zuria (130), West Dembia (55), Takusa (85), and the Wogera district (60). As the total number of health extension workers was less than the sample size we calculated, we included all the health extension workers in these districts.

### 2.5 Study variables

The outcome variable, data management practices, was assessed using 11 questions (38, 39). HEWs who scored above the median score were considered to have good data management practices. HEWs who scored below the median score were considered to have poor data management practices.

The independent variables considered for this study were categorized as follows: sociodemographic characteristics of the HEWs including age, educational status, marital status, work experience, salary, and position; behavioral factors including attitude toward data management practices and knowledge of data management practices; organizational factors including the availability of resources, governance, training, finance, transportation, reference material access, and supportive supervision; and technical factors that affect data management practices including the complexity of data collection tools, the complexity of the report format, and the presence of daily activity challenges.

### 2.6 Data collection tool and quality control

A self-administered, organized, and pre-tested questionnaire was created in English. The data collection process included five supervisors and 20 data collectors. A 3-day training course was provided for the data collectors and a pre-test was conducted outside the study area in West Gondar Zone (Metemma woreda) with a sample size of 10% of the sample of health extension workers in the main study (i.e., 39 health extension workers in Metemma woreda). The validity and reliability of the data collection instrument were assessed using the results of the pre-test. Based on the pre-test results and feedback from area experts, the content of the questionnaire was modified for clarity. Cronbach's alpha reliability test was conducted. A Cronbach's alpha result of 0.71 revealed that the tool was internally consistent and reliable.

### 2.7 Data processing and analysis

Data entry was performed using the EpiData version 4.6 software package and was analyzed using STATA version 16 software. Descriptive statistics were computed to describe the sociodemographic variables. Bivariable and multivariable binary logistic regression analyses were conducted to measure the association between the dependent and independent variables. Variables with a *p*-value less than 0.2 in the bivariable regression analysis were included in the multivariable regression analysis. Multivariable binary logistic regression analysis was conducted to determine the variables significantly associated with the data management practices of health extension workers. The enter method for multivariable binary logistic regression analysis was used to control for the confounding effects of the independent variables. Odds ratios with a 95% confidence level and *p*-value at <0.05 were calculated to ascertain the strength of the association and to indicate statistical significance, respectively. Prior to conducting the logistic regression model, assumptions of multi-collinearity were checked. Furthermore, the multivariable binary logistic regression was fitted using the Hosmer–Lemeshow goodness of fit test.

### 2.8 Ethical consideration

Ethical clearance for this research was obtained from the Institute of Public Health Research Review Ethics Committee at the University of Gondar. A supporting letter was also obtained from the Central Gondar Zonal Health Department. Official letters of cooperation were received from the respective woredas and local health posts. During the data collection, confidentiality and privacy were assured by maintaining the anonymity of participants. Written consent was given by each health professional. The health professionals participated voluntarily and could withdraw from the study at any time if they were not happy with the survey.

## 3 Results

### 3.1 Sociodemographic characteristics of the respondents

Of the 383 participants, all responded to the questionnaire with a response rate of 100%. The mean age of the study participants was 29 years and 84.6% of the respondents were level IV HEWs (Table 1).

**Table 1:** Sociodemographic characteristics of the health extension workers in the Central Gondar Zone, north Ethiopia, in 2021.

Variables	Frequency	Percentage
Age (years)		
18–25	95	24.8
>25	288	75.2
Marital status		
Single	125	32.64
Married	258	67.63
Educational status		15.4
Level III HEW	59	15.4
Level IV HEW	324	84.6
Work experience (years)		32.3
<5	124	32.3
6–10	177	46.2
Above 11	82	21.5
Salary		
Less than 5,358	201	52.5
5,358–8,013	77	20.1
Above 8,013	105	27.4
Position in the health post		
Team leader in the kebele	32	8.4
Coordinator	62	16.2
Health extension worker	289	75.5

### 3.2 Knowledge of data management practices

**Figure 1:**
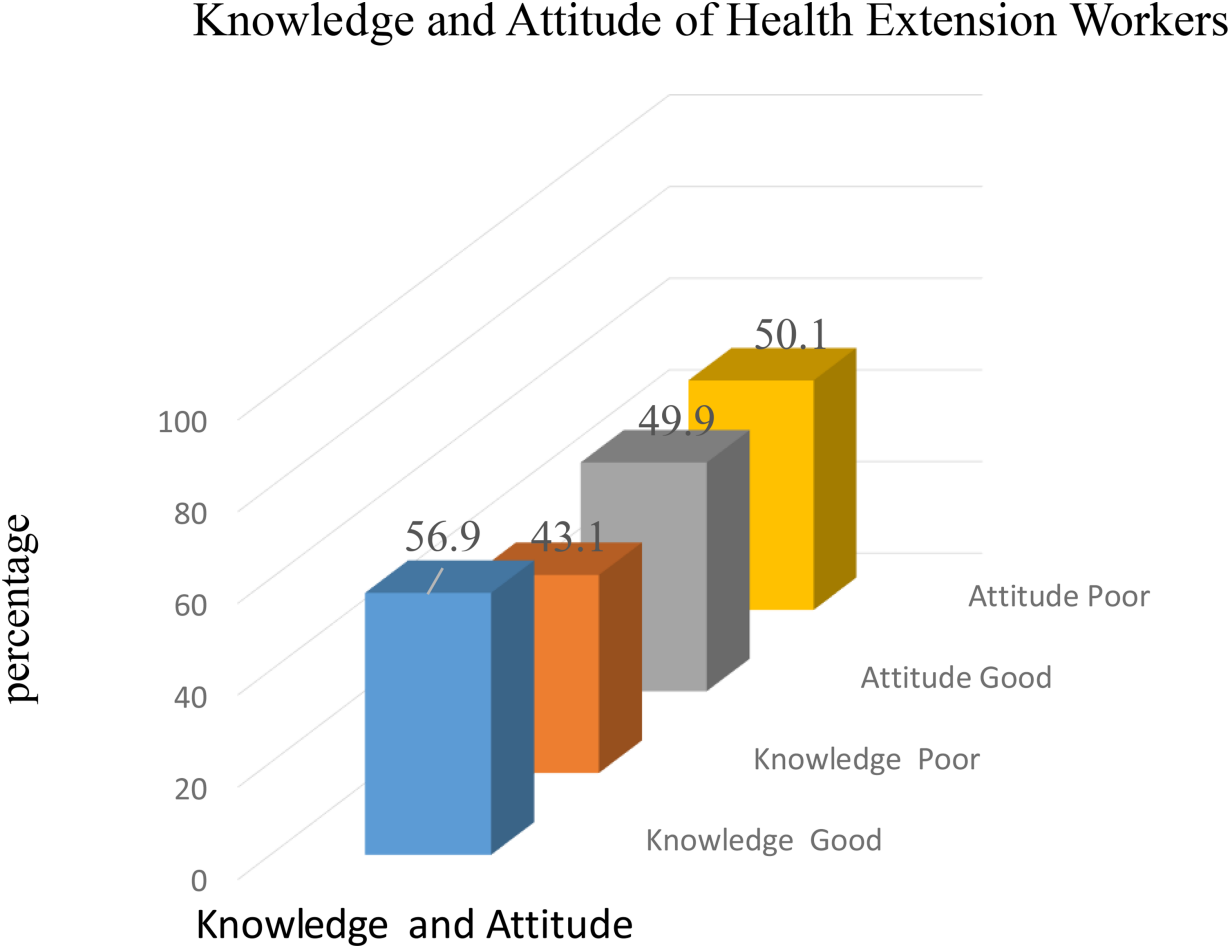
Level of data management knowledge and attitudes among HEWs in the Central Gondar Zone, north Ethiopia.

In total, 320 (83.6%) of the HEWs knew that data management is a procedure that includes data collection and registration. Furthermore, 329 (85.9%) HEWs used data for planning, decision-making, and prevention of disease. The majority of the HEWs, 256 (66.8%), knew to register and tally routine activities reports as a source of data, and 191 (49.9%) and 249 (65%) HEWs knew to ensure accuracy and completeness in data/reports, respectively. Finally, 57% and 49.9% percent of health extension workers had good knowledge of and a good attitude toward data management practices, respectively (Figure 1).

### 3.3 Data management practices of health extension workers

**Figure 2:**
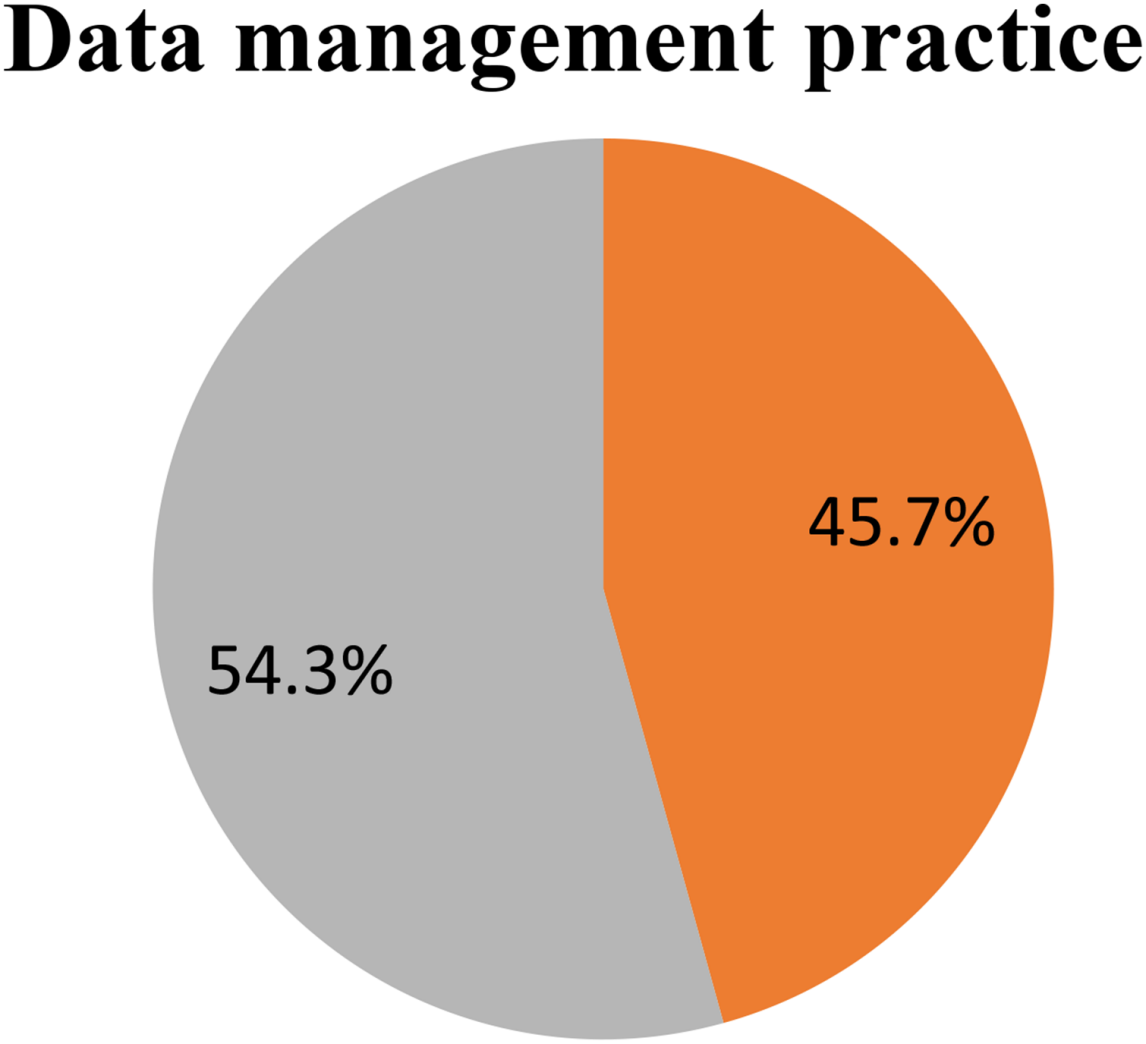
Level of data management practices among health extension workers in the Central Gondar Zone, northwest Ethiopia.

Only 203 (53%) of the HEWs practiced CHIS in their health post according to the required standard. A majority, 291 (76%), correctly registered their daily activities and 246 (64.2%) HEWs used the collected data after converting it into usable information or a report. Finally, 54.3% of the health extension workers had good data management practices (Figure 2).

### 3.4 Technical characteristics of health extension workers

In total, 311 (81.2%) HEWs reported that the registration books, reporting formats, and CHIS materials were easy and simple for them to understand. A majority, 90.9%, responded that they were being challenged by their daily activities (Table 2).

**Table 2:** Technical characteristics related to data management practices in the Central Gondar Zone, northwest Ethiopia, in 2021.

Variables	Frequency	Percentage
CHIS materials are simple and easy		
Yes	311	81.2
No	72	18.7
Complexity of reporting formats		
Yes	30	7.88
No	353	75.9
Challenges in your daily activities		
Yes	348	90.9
No	35	9.1
Daily activity challenges include distance, resistance of community, and complexity of registration		
Yes	218	56.9
No	165	43.1
Overall technical characteristics		
Yes	907	59.2
No	625	40.8

### 3.5 Organizational characteristics of the health extension workers

In total, 310 (80.9%) HEWs had training on data collection, processing, and handling practices, 66 (17.2%) respondents did not have reference material for data management practices in their office, 325 (84.9%) had been supplied with field books, and 315 (82.2%) HEWs had obtained stationery from their respective health office/health center (Table 3).

**Table 3:** Organizational characteristics related to data management practices in the Central Gondar Zone, Northwest Ethiopia, in 2021.

Variables	Frequency	Percentage%
HP has input to strengthen HIS standards		
Yes	165	43.1
No	218	56.9
Attended training on data collection and data handling practice		
Yes	310	80.9
No	73	19.1
Availability of reference materials on CHIS		
Yes	317	82.8
No	66	17.2
Availability of field books		
Yes	325	84.9
No	58	15.1
Availability of reporting formats		
Yes	316	82.5
No	67	17.5
Your cluster HC provides stationery		
Yes	315	82.2
No	68	17.8
Supervision in the past 3 months		
Yes	121	31.6
No	262	68.4
Regularly receive feedback from higher officials		
Yes	123	32.1
No	260	67.9
Incentives for data management		
Yes	124	32.4
No	259	67.6S

HP, health post; HIS, health information system; CHIS, community health information system; HC, health center.

### 3.6 Factors associated with data management practice

The multivariable logistic regression analysis identified the significant factors associated with data management practice. Accordingly, the respondent's attitude, knowledge, work experience, salary, and marital status were significantly associated with their data management practices. Married women were nearly six times (adjusted odd ratio (AOR) = 6, 95% CI = 2.82–12.14) more likely to have good data management practices compared to unmarried women. Health extension workers who had good knowledge of data management practices were 4.7 times more likely to practice good data management (AOR = 4.7, 95% CI = 2.49–8.70). Health extension workers who had a good attitude toward data management practices were six times more likely to have good data management practices (AOR = 6.2, 95% CI = 3.34–11.32). Health extension workers who had 1–5 years of working experience were more likely to have good data management practices (AOR = 4, 95% CI = 1.25–10.5) compared to HEWs who had more than 10 years of working experience. Health extension workers whose monthly income was 5,358–8,013 Ethiopian Birr were 2.7 times more likely to have good data management practices than those who had a monthly salary of less than 5,358 Ethiopian Birr (AOR = 2.7, 95% CI = 1.23–5.95) (Table 4).

**Table 4:** Factors associated with data management practices among health extension workers in the Central Gondar Zone, North Ethiopia, in 2021.

Variables	Data management practice		COR	AOR
	Poor	Good		
Experience				
1–5	77	47	1	1
5–10	90	87	1.58 (0.99–2.53)	3.6 (1.25–10.5)*
Above 10	41	41	1.63 (0.93–2.88)	1.3 (0.61–2.74)
Salary				
<5,358	68	37	1	1
5,358–8,013	93	108	2.13 (1.31–3.47)	2.7 (1.23–5.95)*
Above 8,013	47	30	1.17 (0.64–2.16)	1.5 (0.55–4.21)
User friendliness of formats				
Yes	157	154		1
No	51	18	2.78 (0.65–6.47)	1.5 (0.51–4.27)
Daily activity challenges				
Yes	185	163	1.69 (0.81–3.50)	1.3 (0.43–3.86)
No	23	12	1	1
Inputs to strengthen HIS				
No	135	80	0.46 (0.21–0.70)	0.57 (0.26–1.32)
Yes	72	93		1
Regular feedback				
Yes	55	68	1.77 (1.15–2.73)	1.4 (0.46–4.5)
No	153	107		1
Supportive supervision				
Yes	49	72		1
No	159	103	0.44 (0.28–0.69)	0.4 (0.13–1.06)
Reference material availability				
Yes	148	169	11.4 (4.80–27.20)	1
No	60	6		1.6 (0.16–15.89)
Access to stationery material				
Yes	149	166	7.3 (3.50–15.24)	1.5 (0.33–6.51)
No	59	9		1
Availability of reporting formats				
Ye	150	166	7.13 (3.42–14.89)	1
No	58	9		1.6 (0.25–10.51)
Availability of field books				
Yes	156	169		1
No	52	6	0.1 (0.04–0.26)	0.6 (0.23–14.12)
Training on data collection				
Yes	141	169		1
No	67	6	0.07 (0.03–0.18)	0.2 (0.02–1.53)
Incentives for data management				
Yes	62	62		1
No	143	113	1.26 (0.82–1.94)	2.2 (0.65–7.51)
Attitude				
Poor	144	48	1	1
Good	64	127	5.95 (3.82–9.28)	6.2 (3.34–11.32)**
Knowledge				
Poor	123	42	1	1
Good	85	133	4.58 (2.94–7.14)	4.7 (2.49–8.70)**
Marital status				
Unmarried	90	35	1	1
Married	118	140	3.05(1.92–4.84)	5.9(2.82–12.14)**

COR, crude odd ratio; AOR, adjusted odd ratio; HIS, health information system.

* 0.05 > *p* < 0.01; **0.01 > *p* < 0.001.

## 4 Discussion

Primary healthcare units are major sources of routine healthcare data for the Ethiopian health system since they are in rural settings where approximately 85% of the country's population lives. Healthcare information is important for effective clinical and managerial decision-making at different levels of the healthcare system. Hence, the primary focus of this study was to determine the data management practices of HEWs and their determinant factors.

In this study, only 54.3% of the HEWs had good data management practices. Thus, this could severely affect the decision-making practices of the Ethiopian health system in terms of resource allocation, planning, service quality, and equity. This finding is in line with a study conducted in the East Gojam Zone (38, 39) while it was lower than a study’s finding from Southern Ethiopia (34), in which three-quarters of HEWs had good data management practices. However, the finding of this study is higher compared to another study’s findings in Ethiopia and Palestine (40). This inconsistency is due to differences in data management knowledge such as supervision, study time, community type, and regular feedback. Furthermore, the finding of this study is contrary to a task analysis study conducted in Ethiopia (41), where 97% of the health extension workers perceived themselves to be competent and proficient in performing tasks in all program areas. The wide discrepancy between their data management performance and perception might be due to health extension workers reporting that they do not receive consistent feedback. If there were appropriate and timely feedback, they might understand their performance level better.

The respondent's attitude, knowledge, work experience, salary, and marital status were significantly associated with their data management practices in the Central Gondar Zone.

HEWs who had good data management knowledge were more likely to have good data management practices compared to HEWs who had poor data management knowledge (AOR = 4.7, 95% CI = 2.49–8.70). This finding is supported by different studies conducted in Ethiopia (30, 34, 42, 43) and abroad (44). It is indisputable that good data management knowledge is a prerequisite for good data management practice.

Similarly, HEWs who had a good attitude toward data management practices were six times (AOR = 6, 95% CI = 3.34–11.32) more likely to handle data effectively compared to HEWs who had a poor attitude. Various studies support this finding (8, 21, 26). Thus, attitude is a key factor in the data management practices of HEWs as those who have a good attitude toward data management practices work harder to improve the quality of data and data management practices in their organization.

Married HEWs were nearly six times (AOR = 6, 95% CI = 2.82–12.14) more likely to have good data management practices compared to unmarried HEWs. This could be due to marriage being a key determinant factor for commitment since it is the first step to taking responsibility. Thus, women who are engaged or married have data management practices.

Health extension workers who had 1–5 years of working experience were more likely to have good data management practices (AOR = 4, 95% CI = 1.25–10.5) compared to HEWs who had more than 10 years of working experience. This might be because health extension workers who are more experienced may overwhelmed by the activities they have handled for a long period of time and they may be unmotivated by their lifestyle and working environment.

Finally, health extension workers who had a monthly income of 5,358–8,013 Ethiopian Birr had better data management practices than those who had a monthly salary of less than 5,358 Ethiopian Birr (AOR = 2.7, 95% CI = 1.23–5.95). This could be because a better salary raises a HEW’s motivation to adhere to good data management practices.

## 5 Conclusion

Overall, data management practices were poor with only 54.3% of respondents having good data management practices. Having a good knowledge of data management, availability of field books and registration books, and the understandability of the existing CHIS forms and materials were predictors of good data management practices. The Amhara Regional Health Bureau, in collaboration with Federal Ministry of Health (FMoH), needs to make HMIS/CHIS formats and field notebooks available to health posts. Access to regular data management training and data management information resources for HEWs is also required.

### 5.1 Limitations of the study

The study was not adequately supported by qualitative data. Determining data management knowledge and practice using the median score of knowledge and practice questions may also be a limitation of this study. Furthermore, limited geographic scope, self-reporting bias, a short study period, and the exclusion of health extension workers who are unable to read were limitations of this study.

## Data Availability

The raw data supporting the conclusions of this article will be made available by the authors, without undue reservation.
